# Supplementary material for: Comparing alternative cholera vaccination strategies in Maela refugee camp: using a transmission model in public health practice
Source: BMC Infect Dis. 2019 Dec 21;19:1075. doi: 10.1186/s12879-019-4688-6 (PMC6925891; doi:10.1186/s12879-019-4688-6)
Supplement: Supplementary file 1 — Additional file 1 Appendix. [file 12879_2019_4688_MOESM1_ESM.pdf]

# Appendix

## A1 Profile Likelihoods for Fitted Parameters.

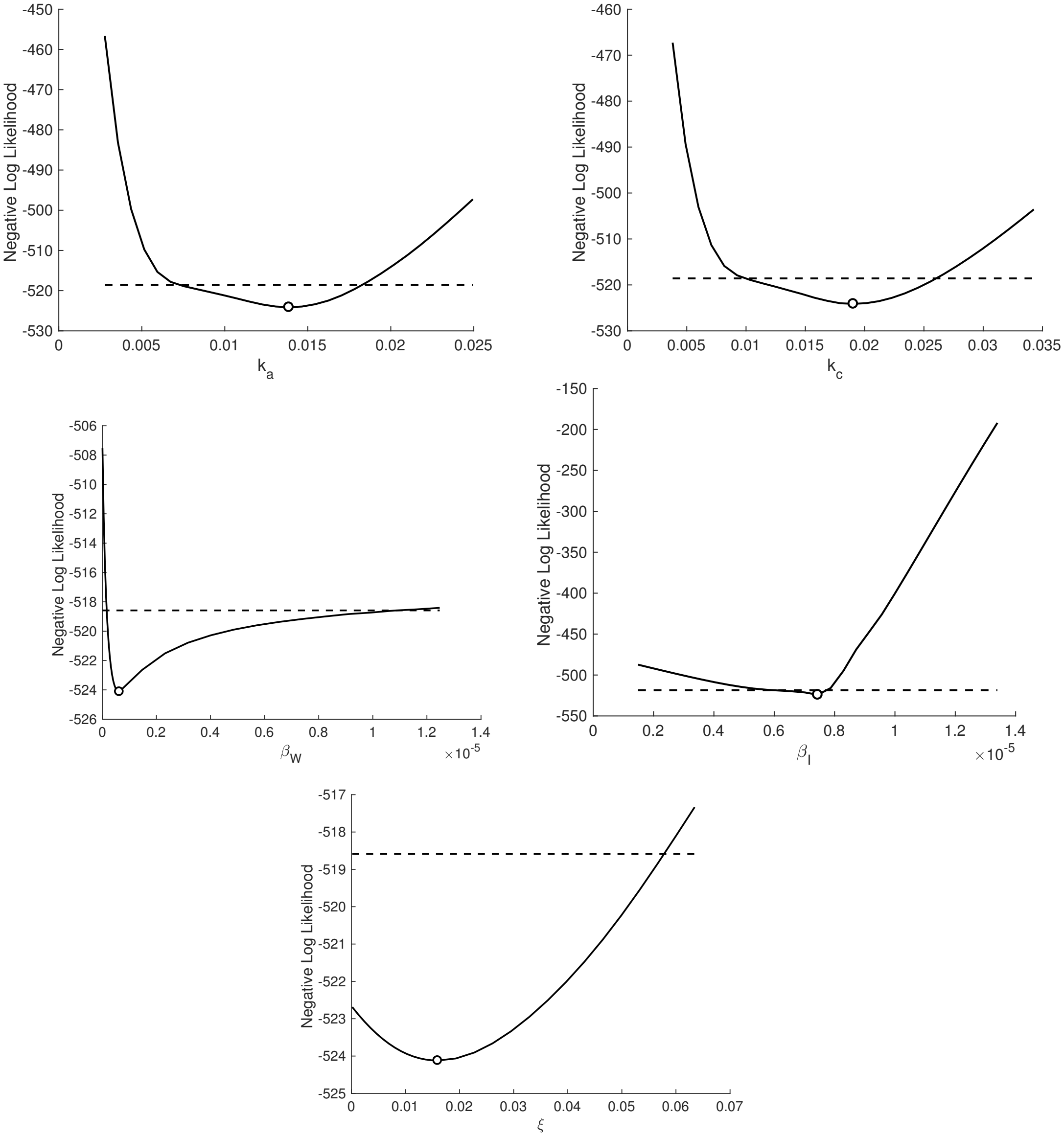


Figure A1: Profile likelihood plots for estimated parameters top row: $k_a$ (left) ,$k_c$ (right), second row: $\beta_W$ (left), $\beta_I$ (right), and third row: $\xi$. Note: The $\beta_W$ and $\xi$ ranges were extended to capture the 95% confidence intervals.

## A2 Cumulative Cases of Alternative Seeding Scenarios.

Table A2: **Cumulative Cases of Alternative Seeding Scenarios.**

| **Vaccination Scenario** | **Cases** | **Attack Rate (per 1,000 people)** |
|---|---|---|
| Baseline: No vaccination | 395.4 | 8.7 |
| Pre-Vaccination | | |
| Maela | 0.1 | 0.002 |
| One-dose only | 239.8 | 5.3 |
| Two-dose only | 233.5 | 5.2 |
| Mixed | 236.6 | 5.2 |
| first come, first served | 237.8 | 5.3 |

## A3 Cumulative Cases of Alternative Seeding Scenarios.

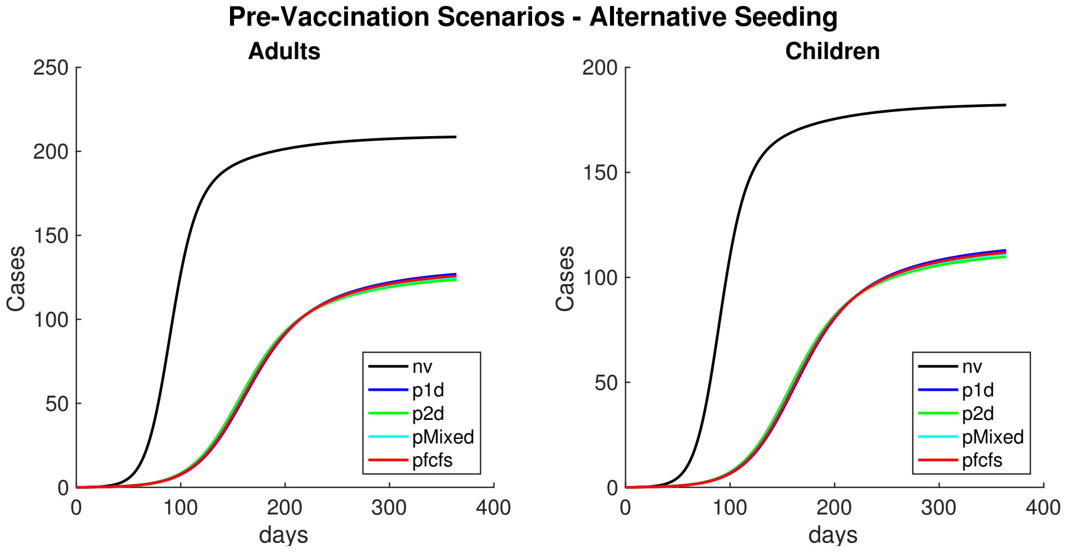


Figure A3: Alternative seeding scenario: one actual case. Cumulative cholera cases in adults and children, for different pre-vaccination scenarios. Where 'nv' is the baseline, no vaccination scenario; 'p1d' is the one-dose scenario; 'p2d' is the two-dose scenario; 'pmixed' is the mixed scenario.

## A4 Maela Vaccination Coverage Calculations.

Table A4: **OCV coverage data from the 2013 vaccine campaign. The "% Coverage" column indicates the percent coverage for the entire population (all included and excluded subjects). The "Number of Individuals" column indicates the initial conditions used in the model, calculated from the coverage percentages.**

| **Class** | **% Coverage** | **Number of Individuals** |
|---|---|---|
| Non-vaccinated infectious adults - seeding ($I_a$) | 0% | 72 |
| Non-vaccinated adults ($S_a$) | 0% | 7,856 |
| Once-vaccinated adults ($V_a$) | 22.3% | 6,208 |
| Twice-vaccinated adults ($VV_a$) | 49.3% | 13,765 |
| Non-vaccinated infectious children - seeding ($I_c$) | 0% | 53 |
| Non-vaccinated children ($S_c$) | 0% | 3,021 |
| Once-vaccinated children ($V_c$) | 18.7% | 3,241 |
| Twice-vaccinated children ($VV_c$) | 63.6% | 11,018 |

# A5 Pre-vaccination with Reduced Vaccine Effectiveness Among Children

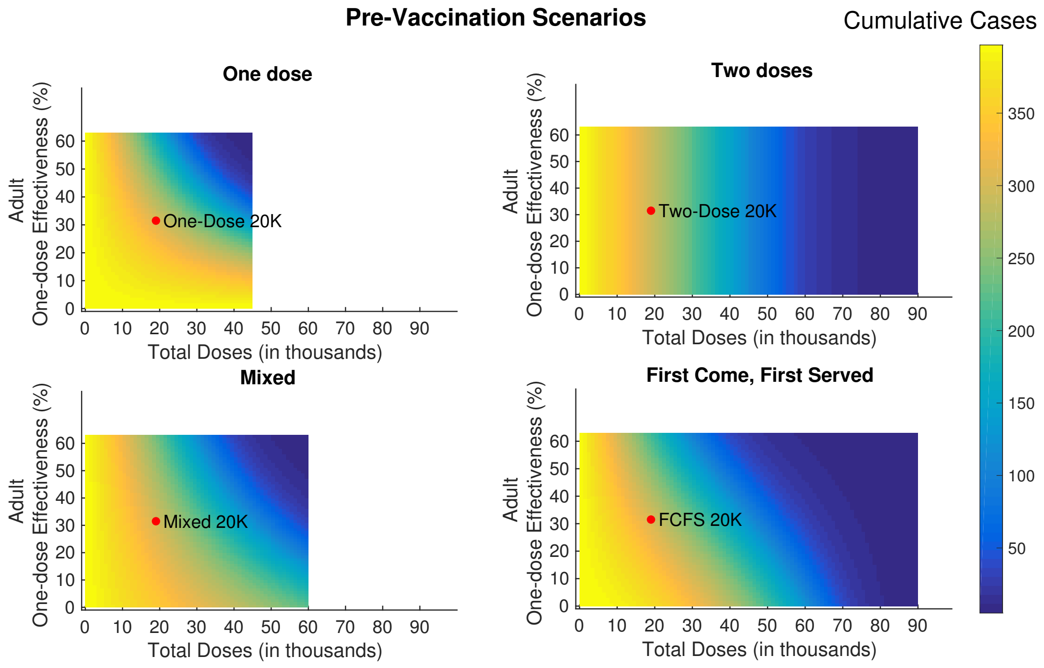


Figure A5: Varying one-dose vaccine effectiveness and total doses for different pre-vaccination scenarios with reduced vaccine effectiveness estimates among children.

## A6 Reactive vaccination with Reduced Vaccine Effectiveness Among Children

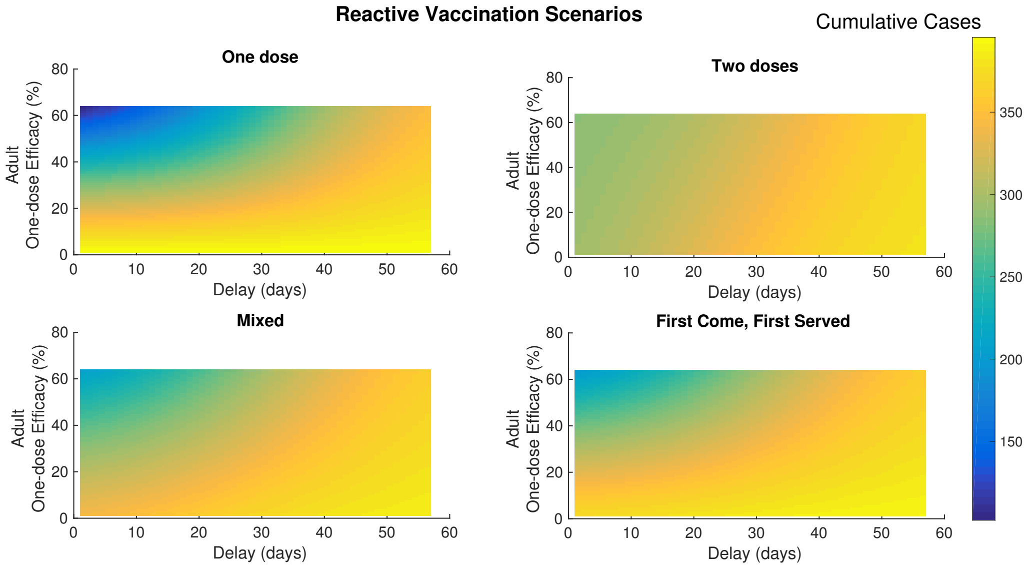


Figure A6: Varying one-dose vaccine effectiveness and delay in campaign implementation for different reactive vaccination scenarios, with reduced vaccine effectiveness estimates among children.

# A7 2013 Forecasting Results with Partially Immune Population.

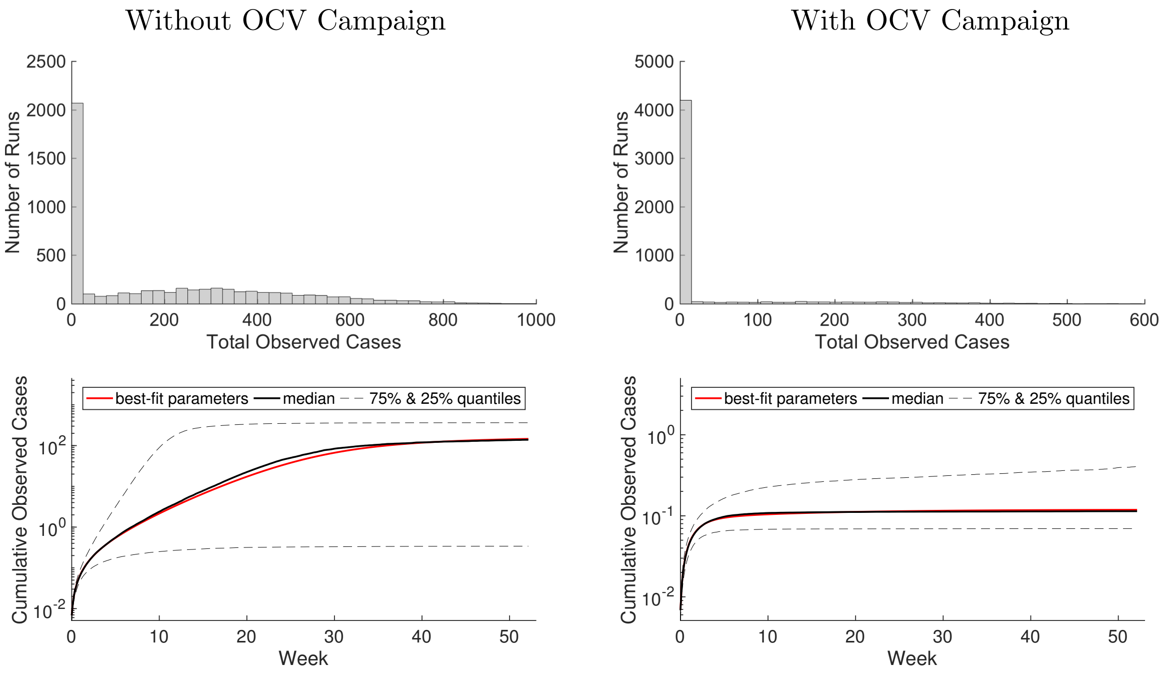


Figure A7: 2013 forecast with a single actual case in adults and children as seeding. These plots show a partially immune population with no OCV campaign on left and with OCV campaign on right.

## A8 2014 Forecasting Results

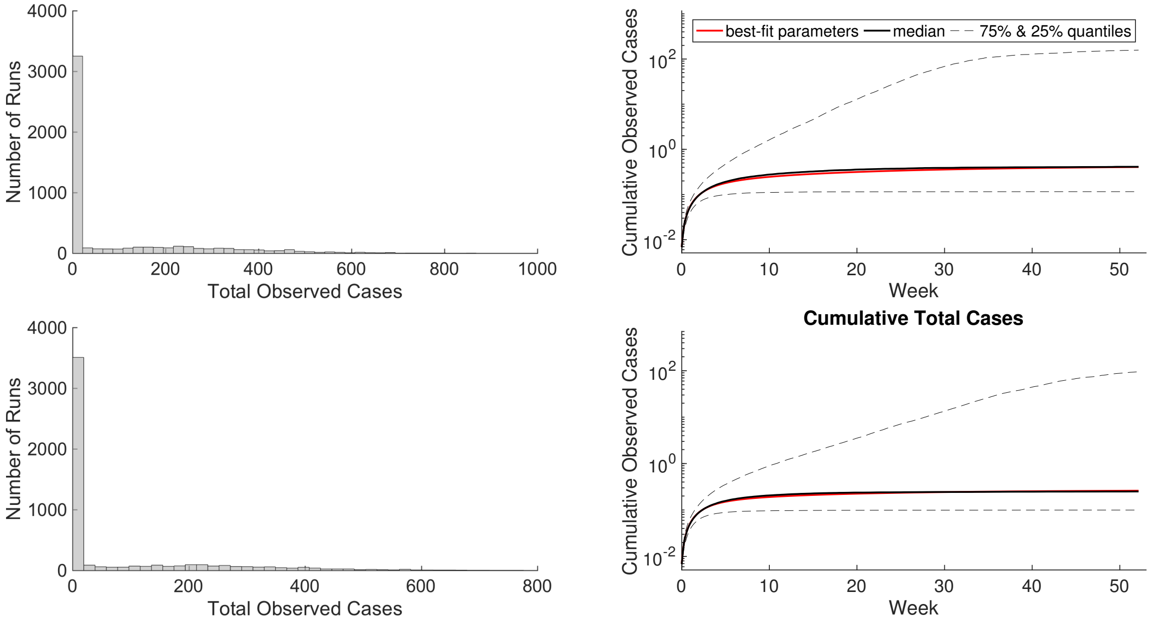


Figure A8: 2014 forecast with a single actual case in adults and children as seeding. First row: Fully susceptible population with OCV campaign. Second row: Partially immune population with OCV campaign.

## A9 Model equations and additional details

### A9.1 Simplified Model Equations

The simplified age-structured model equations with no vaccination are below. This model was used for the identifiability analysis to calculate $\mathcal{R}_0$ and was fit to the Maela outbreak data.

*Force of Infection Equations*

$$\begin{aligned}\lambda_a &= \beta_{aa} I_a + \beta_{ca} I_c + \beta_{wa} W \\ \lambda_c &= \beta_{ac} I_a + \beta_{cc} I_c + \beta_{wc} W\end{aligned} \tag{1}$$

*Non-Vaccinated Adults*

$$\begin{aligned}\dot{S}_a &= \frac{M_a}{2} - \lambda_a S_a - \mu_a S_a \\ \dot{I}_a &= \lambda_a S_a - \gamma I_a - \mu_a I_a \\ \dot{R}_a &= \frac{M_a}{2} + \gamma I_a - \mu_a R_a\end{aligned} \tag{2}$$

*Non-Vaccinated Children*

$$\begin{aligned}\dot{S}_c &= B + \frac{M_c}{2} - \lambda_c S_c - \mu_c S_c \\ \dot{I}_c &= \lambda_c S_c - \gamma I_c - \mu_c I_c \\ \dot{R}_c &= \frac{M_c}{2} + \gamma I_c - \mu_c R_c\end{aligned} \tag{3}$$

*Environmental Pathogen*

$$\begin{aligned}\dot{W} &= \xi(\lambda_w - W) \\ \lambda_w &= I_a + \sigma I_c\end{aligned} \tag{4}$$

### A9.2 Full Model Equations

The full age-structured model equations separated by non-vaccinated, once-vaccinated, and twice-vaccinated individuals are below. If fitted, parameter values are from the simplified model (above) and the remaining non-fitted values (e.g., vaccine effectiveness) are from the literature, see Table 1. This model was used to examine the different counterfactual vaccination scenarios.

*Force of Infection Equations*

$$
\begin{aligned}
\lambda_a &= \beta_{aa}I_a + \beta_{ca}I_c + \beta_{wa}W + \beta_{aa}IV_a + \beta_{ca}IV_c + \beta_{aa}IVV_a + \beta_{ca}IVV_c \\
\lambda_{va} &= (1 - VE_1)(\beta_{aa}I_a + \beta_{ca}I_c + \beta_{wa}W + \beta_{aa}IV_a + \beta_{ca}IV_c + \beta_{aa}IVV_a + \beta_{ca}IVV_c) \\
\lambda_{vva} &= (1 - VE_2)(\beta_{aa}I_a + \beta_{ca}I_c + \beta_{wa}W + \beta_{aa}IV_a + \beta_{ca}IV_c + \beta_{aa}IVV_a + \beta_{ca}IVV_c) \\
\lambda_c &= \beta_{ac}I_a + \beta_{cc}I_c + \beta_{wc}W + \beta_{ac}IV_a + \beta_{cc}IV_c + \beta_{ac}IVV_a + \beta_{cc}IVV_c \\
\lambda_{vc} &= (1 - VE_1)(\beta_{ac}I_a + \beta_{cc}I_c + \beta_{wc}W + \beta_{ac}IV_a + \beta_{cc}IV_c + \beta_{ac}IVV_a + \beta_{cc}IVV_c) \\
\lambda_{vvc} &= (1 - VE_2)(\beta_{ac}I_a + \beta_{cc}I_c + \beta_{wc}W + \beta_{ac}IV_a + \beta_{cc}IV_c + \beta_{ac}IVV_a + \beta_{cc}IVV_c)
\end{aligned}
\tag{5}
$$

*Non-Vaccinated Adults*

$$
\begin{aligned}
\dot{S}_a &= -\mu_a S_a - \lambda_a S_a + M_a/2 - \nu_{a1} S_a \\
\dot{I}_a &= \lambda_a S_a - \mu_a I_a - \gamma I_a \\
\dot{R}_a &= \gamma I_a - \mu_a R_a + M_a/2 - \nu_{a1} R_a
\end{aligned}
\tag{6}
$$

*Once-Vaccinated Adults*

$$
\begin{aligned}
\dot{SV}_a &= -\lambda_{va} SV_a - \mu_a SV_a + \nu_{a1} S_a - \nu_{a2} SV_a \\
\dot{IV}_a &= \lambda_{va} SV_a - \mu_a IV_a - \gamma IV_a \\
\dot{RV}_a &= \gamma IV_a - \mu_a RV_a + \nu_{a1} R_a - \nu_{a2} RV_a
\end{aligned}
\tag{7}
$$

*Twice-Vaccinated Adults*

$$
\begin{aligned}
\dot{SVV}_a &= -\lambda_{vva} SVV_a - \mu_a SVV_a + \nu_{a2} SV_a \\
\dot{IVV}_a &= \lambda_{vva} SVV_a - \mu_a IVV_a - \gamma IVV_a \\
\dot{RVV}_a &= \gamma IVV_a - \mu_a RVV_a + \nu_{a2} RV_a
\end{aligned}
\tag{8}
$$

*Non-Vaccinated Children*

$$
\begin{aligned}
\dot{S}_c &= -\mu_c S_c - \lambda_c S_c + M_c/2 + B - \nu_{c1} S_c \\
\dot{I}_c &= \lambda_c S_c - \mu_c I_c - \gamma I_c \\
\dot{R}_c &= \gamma I_c - \mu_c R_c + M_c/2 - \nu_{c1} R_c
\end{aligned}
\tag{9}
$$

*Once-Vaccinated Children*

$$
\begin{aligned}
\dot{SV}_c &= -\mu_c SV_c - \lambda_{vc} SV_c + \nu_{c1} S_c - \nu_{c2} SV_c \\
\dot{IV}_c &= \lambda_{vc} SV_c - \mu_c IV_c - \gamma IV_c \\
\dot{RV}_c &= \gamma IV_c - \mu_c RV_c + \nu_{c1} R_c - \nu_{c2} RV_c
\end{aligned}
\tag{10}
$$

*Twice-Vaccinated Children*

$$\begin{aligned}
\dot{SVV}_c &= -\lambda_{vvc} SVV_c - \mu_c SVV_c + \nu_{c2} SV_c \\
\dot{IVV}_c &= \lambda_{vvc} SVV_c - \mu_c IVV_c - \gamma IVV_c \\
\dot{RVV}_c &= \gamma IVV_c - \mu_c RVV_c + \nu_{c2} RV_c
\end{aligned} \tag{11}$$

*Environmental Pathogen*

$$\begin{aligned}
\lambda_w &= I_a + \sigma I_c + IV_a + \sigma IV_c + IVV_a + \sigma IVV_c \\
\dot{W} &= \xi(\lambda_w - W)
\end{aligned} \tag{12}$$

*Total Population Sizes*

$$\begin{aligned}
N_a &= S_a + I_a + R_a + SV_a + IV_a + RV_a + SVV_a + IVV_a + RVV_a \\
N_c &= S_c + I_c + R_c + SV_c + IV_c + RV_c + SVV_c + IVV_c + RVV_c
\end{aligned} \tag{13}$$

### A9.3 Forecasting Model equations

The age-structured model equations used for the forecasting scenarios are below.

*Non-Vaccinated Adults*

$$\begin{aligned}
\dot{S}_a &= M_a M_{susc} - \beta_I (I_a + I_c) S_a - \beta_W W S_a + 2\alpha V1_a - \mu_a S_a \\
\dot{I}_a &= (S_a + (1 - VE_{1D}) V1_a + (1 - VE_{2D}) V2_a)(\beta_I (I_a + I_c) + \beta_W W) - \gamma I_a - \mu_a I_a
\end{aligned} \tag{14}$$

*Non-Vaccinated Children*

$$\begin{aligned}
\dot{S}_c &= M_c M_{susc} + B - \beta_I (I_a + I_c) S_c - \beta_W W S_c + 2\alpha V1_c - \mu_c S_c \\
\dot{I}_c &= (S_c + (1 - VE_{1D}) V1_c + (1 - VE_{2D}) V2_c)(\beta_I (I_a + I_c) + \beta_W W) - \gamma I_c - \mu_c I_c
\end{aligned} \tag{15}$$

*Immune Adults*

$$\dot{R}_a = \gamma I_a - 2\alpha R_a - \mu_a R_a \tag{16}$$

*Immune Children*

$$\dot{R}_c = \gamma I_c - 2\alpha R_c - \mu_c R_c \tag{17}$$

*Partial Immune/Vaccinated Adults*

$$\begin{aligned}\dot{V1}_a &= M_a(1 - M_{susc}) - ((1 - VE_{1D})V1_a)(\beta_I(I_a + I_c) - \beta_W W) + \\ &\quad 2\alpha(V2_a - V1_a) - \mu_a V1_a \\ \dot{V2}_a &= ((1 - VE_{2D})V2_a)(-\beta_I(I_a + I_c) - \beta_W W) + 2\alpha(R_a - V2_a) - \mu_a V2_a\end{aligned} \tag{18}$$

*Partial Immune/Vaccinated Children*

$$\begin{aligned}\dot{V1}_c &= M_c(1 - M_{susc}) - ((1 - VE_{1D})V1_c)(\beta_I(I_a + I_c) - \beta_W W) + \\ &\quad 2\alpha(V2_c - V1_c) - \mu_c V1_c \\ \dot{V2}_c &= ((1 - VE_{2D})V2_c)(-\beta_I(I_a + I_c) - \beta_W W) + 2\alpha(R_c - V2_c) - \mu_c V2_c\end{aligned} \tag{19}$$

*Environmental Pathogen*

$$\dot{W} = \xi(I_a + I_c - W) \tag{20}$$

## Identifiability Analysis

Identifiability analysis addresses the question of whether the model parameters can be estimated from a given data set [1]. Identifiability is typically broken into two broad categories—(1) structural identifiability, which examines theoretical identifiability from the structure of the model and measured variables, and (2) practical identifiability, which addresses how a model's identifiability properties are affected by real-world data issues such as noise and sampling frequency.

### Structural Identifiability Analysis

To examine the structural identifiability of our simplified age-structured model, we used the differential-algebra based approach developed in [1–6]. Determining the structural identifiability of the model is a prerequisite to determining if there is a unique solution for a set of unknown model parameters [2]. Structural identifiability can be framed as evaluating whether the model parameters can be estimated uniquely, when the data is assumed to be 'perfect' (i.e., noise-free and measured for all time points). Establishing structural identifiability is a prerequisite for successful parameter estimation from real-world, noisy data. When parameters are not individually identifiable, groups of parameters typically form identifiable combinations that can be uniquely determined.

In the differential algebra approach, the unmeasured state variables (e.g. $S_A$, $S_C$, etc.) are eliminated, leaving equations only the measured variables, their derivatives, and the parameters, denoted the input-output equations. In this case, the measured variables are cholera incidence among adults and children. The identifiability from cholera incidence was more easily analyzed using the prevalence approximation, which as $\gamma$ is assumed to be known, yields the same structural identifiability results as the standard incidence. We assumed the demographic parameters, initial population sizes, and recovery rate are known from data as described above and defined in Table 1, and the remaining parameters ($\beta_{ij}$'s, $k$'s, $\alpha$, $\sigma$, and $\xi$) were considered unknown. A Gröbner-basis

approach was then used to test whether the unknown model parameters in Equations (1) – (4) are identifiable from the measured data, with all calculations performed in Mathematica Version 10.

Similar to the original SIWR model [1], the waterborne transmission parameters and $\alpha$ were not separately identifiable for our model, instead forming the identifiable combination $\bar{\beta}_w = \frac{\alpha}{\xi}\beta_W$. To address this, we define $\bar{W} = \frac{\xi}{\alpha}W$. Rewriting the model equations in terms of these new variables yields the following equation for environmental pathogen:

$$\dot{\bar{W}} = \xi(\lambda_w - \bar{W})$$

with all other equations remaining the same except replacing $W$ with $\bar{W}$ and the identifiable combination $\bar{\beta}_w$. Once re-scaled, all unknown model parameters ($\beta_I$, $\bar{\beta}_W$, $\sigma$, $\xi$, and the $k$'s) were structurally identifiable. From this point forward (and for the parameter estimation and other analyses), we use only the rescaled versions of $\beta_W$ and $W$, and thus we will omit the bar notation.

## Practical Identifiability

Initially, even though structural identifiability was considered, we obtained extremely similar fits for a wide range of transmission parameter values, suggesting that there were practical unidentifiability issues wherein the reporting parameters ($k_a$ and $k_c$) and adult and child transmission parameters can partially compensate for one another to yield the same overall apparent cholera incidences. For the sake of parsimony [7], we set all human-human transmission parameters equal to each other, denoted $\beta_I$, and separately we set all human-water transmission parameters equal to each other, denoted $\beta_W$. Similarly, $\sigma$, the relative shedding rate for adults and children, was also relatively practically unidentifiable, and so we set shedding to be equal for both classes.

To examine practical identifiability and parameter uncertainty, we plotted profile likelihoods of each fitted parameter. Profile likelihoods are a numerical approach to evaluating parameter uncertainty and identifiability [8]. Profiles are generated by fixing the profiled parameter to a series of values, while fitting the remaining parameters that are being estimated. Typically, the minimum negative log likelihood (or equivalently the maximum likelihood) values are plotted for each value of the profiled parameter, forming the profile likelihood for that parameter. The minimum represents the best-fit value of the profiled parameter and is determined by parameter estimation. If the profile is flat, the parameter cannot be uniquely determined and is considered unidentifiable. However, even if the profile is structurally identifiable, the curvature may be quite shallow, so that a particular minimum cannot practically be distinguished - this is denoted practical unidentifiability. Confidence intervals can be determined from the profile likelihood by setting a significance-based threshold on the likelihood based on a $\chi^2$ distribution [8]. Once the threshold is set, all parameters corresponding to likelihood values below the threshold fall within the confidence interval. The results from the profile likelihood plotting can be seen in Figure A1.

## Sensitivity Analysis: Initial Seeding from Observed to Actual

As another sensitivity analysis, we changed our initial seeding from one observed case to one actual case for the Maela and pre-vaccination scenarios. Because vaccination occurred before any outbreak, administration of the vaccine was not affected by case detection. Overall, we see the same pattern of results, shown in Figure A3 and Table A2. The two-dose scenario sees the largest reduction in cases followed by the mixed, first come, first served, and one-dose scenarios. Since the number of initial infected individuals is lower, the total cumulative case counts are as well. Of note is that the reduction in cases is greater for pre-vaccination scenarios than for the baseline non-vaccination scenario.

## Maela Vaccine Coverage Calculations

Among included individuals (pregnant women and infants $<$ 1 year were excluded), the OCV campaign covered 51% of adults and 68% of children with two doses, and another 23% of adults and 20% of children with one dose. We made the following adjustments to determine total Maela coverage among both included and excluded individuals for the forecasting scenarios:

- Once-vaccinated adults:
  $(V_a - VV_a)((N_a-$ Pregnant women$)/(N_a))N_a$
  ((0.74-0.51)*(27901-910)/27901)*27901 = **6207.9**
- Twice-vaccinated adults:
  $(VV_a)((N_a-$ Pregnant women$)/(N_a))N_a$
  (0.51*(27901-910)/27901)*27901 = **13765.4**
- Once-vaccinated children:
  $(V_c - VV_c)((N_c-$ infants under 1 year old$)/(N_c))N_c$
  ((0.88-0.68)*(17332-1129)/17332)*17332 = **3240.6**
- Twice-vaccinated adults:
  $(VV_c)((N_c-$ infants under 1 year old$)/(N_c))N_c$
  (0.68*(17332-1129)/17332)*17332 = **11018**

Table A4 shows the total number of individuals by class used to simulate the OCV campaign.

### Forecasts for the 2013 Cholera Season

In the 2013 forecasting results, we see a larger spread of total case numbers for runs in the scenario without the OCV campaign compared to the scenario with the OCV campaign. The partially immune population runs generally have lower case counts when comparing to the fully susceptible population. Furthermore, for the scenarios that consider the OCV campaign, we see that the vast majority of runs having case counts close to 0. For details see Figures 8 and A7.

### Forecasts for the 2014 Cholera Season

The 2014 forecasting results are quite similar to the 2013 runs for the fully susceptible population compared to the partially immune population with more runs resulting in 0 total cases for the partially immune population. As population immunity wanes between 2013 and 2014 we get a higher proportion of larger outbreaks for the 2014 forecasting scenarios, but the vast majority of runs remain close to 0 for both the fully susceptible and partially immune populations. For details see Figure A8.

## References


[1] Eisenberg MC, Robertson SL, Tien JH. Identifiability and estimation of multiple transmission pathways in cholera and waterborne disease. Journal of Theoretical Biology. 2013;324:84–102.

[2] Audoly S, Bellu G, D'Angio L, Saccomani MP, Cobelli C. Global identifiability of nonlinear models of biological systems. IEEE Transactions on Biomedical Engineering. 2001;48(1):55–65.

[3] Evans ND, White LJ, Chapman MJ, Godfrey KR, Chappell MJ. The structural identifiability of the susceptible infected recovered model with seasonal forcing. Mathematical Biosciences. 2005;194(2):175–197.

[4] Chapman JD, Evans ND. The structural identifiability of susceptible–infective–recovered type epidemic models with incomplete immunity and birth targeted vaccination. Biomedical Signal Processing and Control. 2009;4(4):278–284.

[5] Bellman R, Åström KJ. On structural identifiability. Mathematical Biosciences. 1970;7(3-4):329–339.

[6] Chis OT, Banga JR, Balsa-Canto E. Structural identifiability of systems biology models: a critical comparison of methods. PloS One. 2011;6(11):e27755.

[7] Stoica P, SÖDERSTRÖM T. On the parsimony principle. International Journal of Control. 1982;36(3):409–418.

[8] Raue A, Kreutz C, Maiwald T, Bachmann J, Schilling M, Klingmüller U, et al. Structural and practical identifiability analysis of partially observed dynamical models by exploiting the profile likelihood. Bioinformatics. 2009;25(15):1923–1929.
